# Factors Affecting the Delivery and Acceptability of the ROWTATE Telehealth Vocational Rehabilitation Intervention for Traumatic Injury Survivors: A Mixed-Methods Study

**DOI:** 10.3390/ijerph18189744

**Published:** 2021-09-16

**Authors:** Jade Kettlewell, Rebecca Lindley, Kate Radford, Priya Patel, Kay Bridger, Blerina Kellezi, Stephen Timmons, Isabel Andrews, Stephen Fallon, Natasha Lannin, Jain Holmes, Denise Kendrick, on behalf of the ROWTATE Team

**Affiliations:** 1 Centre for Health Innovation, Leadership & Learning, Business School, University of Nottingham, Nottingham NG8 1BB, UK; Stephen.Timmons@nottingham.ac.uk; 2 School of Medicine, University of Nottingham, Nottingham NG7 2UH, UK; Rebecca.lindley@nottingham.ac.uk (R.L.); Kate.radford@nottingham.ac.uk (K.R.); Kay.bridger@nottingham.ac.uk (K.B.); Blerina.kellezi@ntu.ac.uk (B.K.); issyandrews@hotmail.com (I.A.); fallon@btinternet.com (S.F.); Jain.holmes@nottingham.ac.uk (J.H.); Denise.kendrick@nottingham.ac.uk (D.K.); 3 Institute of Mental Health, University of Nottingham, Nottingham NG7 2TU, UK; mszpp@nottingham.ac.uk; 4 Department of Psychology, Nottingham Trent University, Nottingham NG1 4FQ, UK; 5 Department of Neuroscience, Monash University, Melbourne, VIC 3004, Australia; Natasha.Lannin@monash.edu

**Keywords:** traumatic injuries, return to work, vocational rehabilitation, patient perspectives, telehealth, acceptability, mixed methods, occupational therapy, clinical psychology

## Abstract

Background: Returning to work after traumatic injury can be problematic. We developed a vocational telerehabilitation (VR) intervention for trauma survivors, delivered by trained occupational therapists (OTs) and clinical psychologists (CPs), and explored factors affecting delivery and acceptability in a feasibility study. Methods: Surveys pre- (5 OTs, 2 CPs) and post-training (3 OTs, 1 CP); interviews pre- (5 OTs, 2 CPs) and post-intervention (4 trauma survivors, 4 OTs, 2 CPs). Mean survey scores for 14 theoretical domains identified telerehabilitation barriers (score ≤ 3.5) and facilitators (score ≥ 5). Interviews were transcribed and thematically analysed. Results: Surveys: pre-training, the only barrier was therapists’ intentions to use telerehabilitation (mean = 3.40 ± 0.23), post-training, 13/14 domains were facilitators. Interviews: barriers/facilitators included environmental context/resources (e.g., technology, patient engagement, privacy/disruptions, travel and access); beliefs about capabilities (e.g., building rapport, complex assessments, knowledge/confidence, third-party feedback and communication style); optimism (e.g., impossible assessments, novel working methods, perceived importance and patient/therapist reluctance) and social/professional role/identity (e.g., therapeutic methods). Training and experience of intervention delivery addressed some barriers and increased facilitators. The intervention was acceptable to trauma survivors and therapists. Conclusion: Despite training and experience in intervention delivery, some barriers remained. Providing some face-to-face delivery where necessary may address certain barriers, but strategies are required to address other barriers.

## 1. Introduction

Traumatic injuries are a major public health problem for working age adults [1]. Improved survival rates [2] result in a greater number of people living with the long-term physical and psychological effects of injury with impacts on return to work. Systematic reviews have found that up to 2 years post-injury, only 41% of people with traumatic brain injury [3], 21–67% with spinal cord injury [4] and 42–85% with orthopaedic injuries [5] had returned to employment. This suggests that following traumatic injuries, many people may require support to return to the workplace.

Vocational rehabilitation (VR) can be defined as ‘a multi-professional approach that is provided to individuals of working age with health-related impairments, limitations, or restrictions with work functioning and whose primary aim is to optimise work participation’ [6]. VR interventions have been shown to be effective in helping injured patients return to work, although the evidence mainly focuses on brain and spinal cord injury [7,8,9]. Over recent years, there has been increasing interest in providing rehabilitation using telecommunication-based practices (telerehabilitation). Telerehabilitation has been shown to overcome issues including access to specialist services [10,11], and patient cost and travel time [12,13], which often disproportionally affect people living in rural areas or a long distance from major trauma centres [14,15]. Telerehabilitation has been used successfully with trauma survivors, for example, remote interventions for brain injury patients to improve physical activity [16], fatigue [17], communication skills [18], depression [19] and emotional regulation [20].

A small number of studies report work outcomes following telerehabilitation for those with brain or spinal cord injury. One randomised controlled trial (RCT) investigated provision of electronic job-searching and career planning modules for spinal cord injury patients [21], finding no significant effect on job-seeking self-efficacy, but significant improvements in optimism levels. A second RCT [22] found no significant effect on return-to-work rates in patients with mild traumatic brain injury receiving telephone counselling as opposed to face-to-face cognitive behavioural therapy. Many unanswered questions remain regarding optimal telerehabilitation interventions and the impact of telerehabilitation on return to work amongst a general trauma population.

Barriers to telerehabilitation have been identified in the broader literature, including technical problems [10,18,23,24,25,26], patient difficulty in using the necessary technology [27,28], difficulty conducting physical examinations [10,12,15,29] or psychosocial components [30], communication problems [31,32] and provider resistance to adopt telerehabilitation [27,29]. Some studies have found that patients prefer telerehabilitation to face-to-face rehabilitation, with others suggesting that patients have no preference for either mode [31,32,33].

As part of a larger programme of research (National Institute for Health Research (NIHR) funded; ROWTATE study) to develop and test the effectiveness and cost-effectiveness of a remote VR intervention for trauma patients with a wide range of injuries (hereafter referred to as general trauma patients), this study explores factors affecting delivery and acceptability of the intervention within a feasibility study [34]. Our VR intervention was originally designed for face-to-face delivery, but as a result of the global COVID-19 pandemic in 2020, many rehabilitation interventions had to rapidly adapt to virtual delivery. We therefore adapted the content of our VR intervention for remote delivery, drawing on existing evidence and expertise of colleagues (NL). We subsequently adapted our feasibility study and therapist training package.

Our intervention provides individually tailored VR, predominantly by video call or telephone [14], delivered by occupational therapists (OTs) acting as case-coordinators with referral to clinical psychologists, where necessary. The intervention begins within 12 weeks post-injury and lasts up to 12 months. It involves assessing the impact of the injury on the person and their work, rehabilitation to prepare them for work, and plan and monitor a phased return to work. It also involves liaison with employers, the health care team and solicitors; negotiating workplace adaptions, and educating patients and employers about the impact of injury on work. The psychological component of the intervention provides early identification, monitoring and support for psychological problems, with referral to a clinical psychologist where necessary. Occupational therapists and clinical psychologists are required to work as a team to develop and use case formulation to inform patient goals and intervention delivery.

The aims of this study were to: (1) identify barriers and facilitators to delivery of a remote VR intervention for general trauma patients and (2) explore acceptability of the VR intervention to patients and their occupational therapists, clinical psychologists and employers.

## 2. Methods

This study formed part of a mixed-methods feasibility study, and addressed specific feasibility study objectives, which are shown in Table 1. The findings from this study have informed intervention development, therapist training and trial methodology ahead of a definitive RCT. Ethical approval was obtained from the North of Scotland NHS Research Ethics Committee (Ref: 19/NS/0130). Verbal informed consent was obtained from interviewees.

Occupational therapists and clinical psychologists were trained by expert vocational therapists (author JH and a clinical neuropsychologist) and the research team in remote delivery of the intervention. All therapists (n = 5 occupational therapists, n = 2 clinical psychologists) trained to deliver the intervention were invited via email to complete a survey before and within one month of completing training and interviews before and approximately three months after starting intervention delivery. The latter time frame was chosen to allow enough time for therapists to have delivered at least two intervention sessions and made contact with their patient’s employer, where appropriate. All trauma survivor participants (n = 10) were invited via video, telephone or email to an interview approximately three months after starting the intervention. All interviewed trauma survivor participants who were employed were asked for consent to invite their employers to an interview. Interviewees were provided with information about the study aims in a participant information sheet.

The survey measured behavioural determinants acting as barriers and facilitators to implementing remote VR interventions. It was informed by the theoretical domains framework (TDF), which integrates 33 behaviour change theories and consists of 14 domains describing behaviour determinants (see Table 2 for domains). We used the TDF as it is a validated framework that has been used to identify implementation problems and professional behaviours as a basis for intervention development [35]. Each TDF statement was scored on 7-point Likert scale (7 = strongly agree, 1 = strongly disagree). Mean and standard deviation TDF domain scores were calculated. Mean domain scores of ≤3.5 indicated substantial barriers and mean domain scores of ≥5 indicated facilitators to implementing the remote VR intervention. Descriptive statistics were used to describe domain scores. Statistical comparisons were not made due to the small number of observations. The surveys are provided as Supplementary Files S1 and S2.

Pre- and post-intervention interview topic guides were informed by a review of the telerehabilitation literature and the TDF. The post-intervention topic guide was also informed by the Theoretical Framework of Acceptability (TFA) [36]. In pre-intervention interviews, therapists were asked about their views on the remote delivery of VR and telerehabilitation, indicating potential barriers and facilitators for implementation. During post-intervention interviews, therapists and trauma survivors were asked about acceptability of the VR intervention, and barriers and facilitators to remote delivery. Topic guides are provided as Supplementary Files 3–5. Interviews were conducted by four authors (JK, RL, PP, KB). JK has a PhD and was working as a post-doctoral research fellow, with expertise in rehabilitation, implementation and qualitative research. RL, PP and KB have a MSc with a background in psychology and/or rehabilitation, all working as research assistants. RL, PP and KB were trained to conduct the interviews by JK and ST (professor of health services management with extensive qualitative experience) and supervised by JK. Patient interviews were pilot tested with members of the study patient and public involvement (PPI) group.

Interviews were conducted between August 2020 and March 2021, via Microsoft Teams or by telephone, lasting up to one hour, all were audio recorded and then transcribed. Interviewers already knew the therapists prior to conducting the interviews, as they had attended intervention training prior to this. One interviewer (RL) had previously spoken to one trauma survivor participant prior to the interview, as she had recruited them to take part in the associated feasibility study. Interviewees were also aware that the researchers were developing the intervention for delivery in a definitive trial.

Interview transcripts were independently coded by four authors (JK, RL, PP, KB) using NVivo. Framework analysis was used to analyse the data, informed by the TDF and TFA [37]. Five steps were used: transcription, familiarisation, coding, developing framework and applying framework. Main themes were agreed by discussion between researchers. Two trauma survivors and PPI authors (IA, SF) were involved in analysing and interpreting patient data. IA and SF were trained to conduct interview analysis by ST and JK. The final framework (Table 2) was developed and discussed with other authors for agreement.

## 3. Results

Seven therapists completed the pre-training survey (n = 5 occupational therapists, n = 2 clinical psychologists) and four completed the post-training survey (n = 3 occupational therapists, n = 1 clinical psychologist), due to study withdrawal (n = 1), declining survey completion (n = 1) and non-response (n = 1).

Pre-intervention interviews were conducted with all seven therapists delivering the intervention and six therapists were interviewed post-intervention. Therapist characteristics are shown in Table 3. Four patients were interviewed post-intervention. No employer interviews were conducted. One patient was self-employed, two declined consent for employer contact and one employer did not respond to the study invite. Patient participant characteristics are shown in Table 4.

### 3.1. Quantitative Data

Pre- and post-training survey data are shown in Table 5, including the individual items that comprise each domain. Pre-training, therapists’ intentions to use telerehabilitation was the only domain identified as a substantial barrier) and two facilitators were identified: social influences and knowledge. These barriers and facilitators informed design of the occupational therapist/clinical psychologist training and of the intervention. Post-training 13 of the 14 domains were identified as facilitators. The highest scoring domains were social/professional role or identity, knowledge and social influences. The only domain not scoring as a facilitator was memory, attention and decision processing.

### 3.2. Qualitative Data

**Figure 1:**
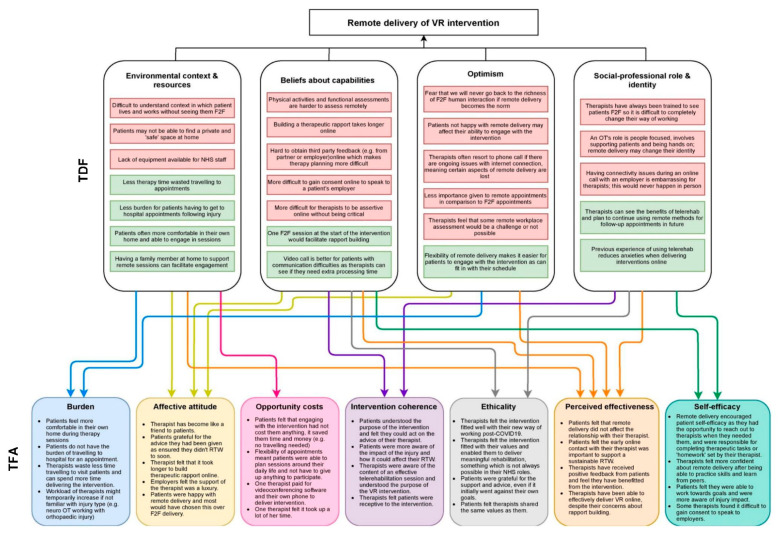
**Summary of interview themes mapped onto the TDF and TFA.** F2F: face to face; OT: occupational therapist; TDF: theoretical domains framework; TFA: Theoretical Framework of Acceptability; VR: vocational rehabilitation. Red boxes in the TDF section refer to barriers to remote delivery of VR and green boxes in the TDF section refer to facilitators to remote delivery of VR. Arrows between TDF and TFA boxes indicate influences on acceptability.

Interviews identified barriers and facilitators to remote delivery of the VR intervention and factors contributing to acceptability. The key themes related to barriers and facilitators were: environmental context and resources; beliefs about capabilities; optimism and social/professional role and identity. A visual summary of barriers and facilitators to remote delivery, and acceptability of the intervention is shown in Figure 1.

#### 3.2.1. Pre-Intervention Interviews

##### Environmental Context and Resources

Access to technologies to enable telerehabilitation was the main barrier identified in this theme by therapists. Laptop shortages required therapists to share laptops, drive to and from the hospital to use laptops and limited options for booking patient appointments. NHS Trust policies precluded therapists from using personal computers. The lack of equipment caused tension within therapy teams:

*‘* *When we’ve been doing video conferencing since April, it’s bonkers that we haven’t got enough computers. I mean it’s daft when you’re in the unit to do video calls. It’s, “Well, I need a laptop.” “I’m doing a video call.” So we actually have to use a diary, ridiculously, to make sure there’s no more than two of us trying to do a video call at once because we haven’t got any more laptops to do it from.’*

(OT03)

Therapists felt that remote delivery could make it difficult to assess a patient’s environment and understand the context in which they live without seeing them face to face.

*‘* *If it’s a home visit and you’re seeing somebody in their own environment, you can get a sense of that [context]… get a sense of how people are living and observe where they’re at in ways that you wouldn’t get [remotely].’*

(CP06)

Some therapists voiced concerns regarding privacy and disruptions:

*‘* *It’s whether there’s a room available and space available. So yeah, that becomes tricky. And obviously you need that private space, don’t you, to do the video calling? You can’t just do that anywhere, and for privacy for the patient, they need that confidentiality that you’re in a room on your own…room usage is a real issue.’*

(OT03)

Therapists highlighted potential benefits of a remote VR intervention, including reduced travel time for therapists and patients, allowing more therapy sessions to be delivered and increased flexibility with how interventions can be delivered:

*‘* *You can fit it [therapy session] in around your working week… maybe you could check in with someone a little bit more often rather than waiting for that face-to-face appointment.’*

(CP02)

Therapists also suggested that having someone available to support the patient (a technology support partner) may improve remote therapy sessions by helping positioning the device or holding it while the patient completes a therapy task.

##### Beliefs about Capabilities

A range of barriers related to capabilities were identified. Therapists felt that it would be difficult to conduct more complex cognitive and mood assessments remotely:

*‘* *I think if you’re wanting to do more complex cognitive assessments, I think that might be a bit more challenging, particularly with wanting to see how the person approaches the task and you’re more interested in that than the actual answers and score.’*

(OT07)

Several therapists, reflecting on prior experiences of delivering telerehabilitation, had concerns about building therapeutic rapport or engaging with patients on an emotional level:

*‘* *I just couldn’t get the rapport because he’s [patient] so low. He’s low, he’s anxious, he’s fed up, he just says, “I don’t know,” all the time. I just couldn’t get anywhere with a telephone call with him, and now I’ve started to get somewhere. He’s one of the ones I’ve prioritised as face to face because work were going to kick him out…now I’m seeing him face to face it’s completely different.’*

(OT03)

Some therapists felt they lacked knowledge or confidence in delivering telerehabilitation and that training and learning from colleagues may help address this:

*‘* *I think if I have sufficient training and maybe problem solving and group work in terms of learning from others how they have done it so far, then—and maybe it will increase knowledge and skill and that, it will improve even more.’*

(OT04)

Rapid adaptations as a result of COVID-19 resulted in all therapy being remotely delivered and things that seemed impossible prior to this, were suddenly possible (e.g., conducting remote functional assessments):

*‘* *I think when we first started doing it [remote delivery] we were a bit like, oh God, what can I do on a video call? Because if you’d asked me six months ago, “Could you do some of your job on video calls?” I’d be like, “No, I need to see patients face-to-face. Don’t be ridiculous”…“Of course I can’t do hand function [assessments]. I can’t touch their hands,” but you’ve got a relative next to them, they’re showing you the [movement] range, or if you’ve got a really good carer, you might be able to do some of that. So, we’ve evolved hugely in what we think we can do, and that’s definitely from listening to each other.’*

(OT03)

Although therapists raised several barriers to telerehabilitation, they believed that providing remote VR to trauma survivors would work in the context of this study. Gaining some telerehabilitation experience in their normal clinical roles and learning from other colleagues would facilitate its delivery.

*‘* *I think I’d be a lot more confident having had experience of delivering it with stroke patients. And certainly I’ve learned stuff from my training as well, or we’ve been learning together about how to deliver kind of more—slightly more complex kind of information.’*

(CP02)

##### Optimism

Some therapists felt that it would be impossible to conduct some workplace assessments remotely, as it would limit their understanding of the work environment without seeing it in person, particularly for non-office-based occupations:

*‘* *If you’re going to have someone that is more in construction, I think that is impossible because no one’s going to walk around with an iPad and tell you exactly. And you won’t be able to do a physical assessment in terms of the space and what it does entail and everything like that.’*

(OT04)

Therapists believed that patient confidence in using technology influenced their acceptance:

*‘* *Then the other issues are just about whether the participants, the patients, will have the right technology to be able to take part…I can imagine for individuals—thinking about people I’ve worked with in the past—there can be a real reticence or some concern about doing things remotely.’*

(CP06)

Others suggested that trauma survivors might have had enough of online interaction (as a result of COVID-19) and fear that things may never return to ‘normal’:

*‘* *Everybody’s had enough of not seeing people face to face. It’s just not human…I fear that the more work we all do to adapt things, the more likely it is that we’re never going to go back to how it was before…that’s my key worry about all of this, is that at some point it may just be that we never go back to having the richness of human involvement.’*

(OT05)

However, most therapists were positive about therapy moving towards remote delivery and how changes in the health care system had been revolutionary and beneficial:

*‘* *It’s good because I think it probably has revolutionised how we’re doing things.’*

(OT07)

*‘* *Follow-up appointments would become a lot easier, I guess. I’m not sure I’d love to be doing initials over a video call for ever and a day, but then it serves its place. If you’ve got patients that are miles away, it still has its place…there are certain ones that I think lend themselves really well and, yeah, why wouldn’t you do a video call in the future?’*

(OT03)

##### Social/Professional Role and Identity

One of the main barriers for therapists was the change in professional role and identity. Therapists have been trained to deliver interventions face to face and their work has always focused on seeing patients in person, assessing them, reading body language and understanding their environment. Many mentioned how challenging moving away from traditional therapeutic methods has been:

*‘* *That’s been a challenge for me as a psychologist I think in providing that therapeutic relationship because it’s—my entire training, my entire working life has been based on face-to-face contact’*

(CP02)

#### 3.2.2. Post-Intervention Interviews

##### Environmental Context and Resources

Post-intervention interviews considered factors affecting intervention delivery and acceptability of remote VR. Trauma survivors mentioned few barriers to remote delivery. One mentioned remote interventions might be difficult for people unfamiliar with IT or without reliable internet connection:

*‘* *I’m not sure how that would be for someone who perhaps doesn’t work that much with IT in their daily work as I do. So I’m quite aware that it may well be much more of a problem for someone who perhaps has no Wi-Fi at home and would have to rely on mobile reception.’*

(Trauma survivor, polytrauma)

One patient suggested that remote intervention delivery may pose problems for those with brain injuries or fatigue, as concentrating on a screen for a long period of time is difficult:

*‘* *You know when like we’re talking at a camera, like an hour and a half, I mean I was falling asleep if I concentrated on something for an hour as it was…like I was absolutely wasted during these [video call] things.’*

(Trauma survivor, traumatic brain/neck injury)

Although therapists were generally positive about the intervention, several of the barriers identified in the pre-intervention interviews remained. Therapists still felt it was more difficult to understand the context of where a trauma survivor lives and works without visiting them in person and they still had concerns about privacy and distractions for those individuals:

*‘* *I would have liked to have met my clients once face to face. I think it would have given me more idea of the context in which they lived and worked. And I do think it enhances the therapeutic relationship.’*

(OT05)

Therapists highlighted a new barrier, relating to rapidly changing NHS Trust policies about software platforms they were permitted to use for video calls. Having been trained to use one platform, they were subsequently only allowed to use a different platform:

*‘* *Learning, for me, is easier if you show me how to do it and then I’m straight onto practising to do it. But actually, because of [NHS Trust’s] rules, we were having to use DrDoctor [video call platform]. So, you kind of learn all this detail [previous software platform] and then go, right, anyway, park that because you’re doing DrDoctor, and that’s your only option.’*

(OT03)

Several facilitators were identified by trauma survivors and therapists. Similar to pre-intervention interviews, minimising time spent travelling to hospital appointments was seen as a key benefit of remote delivery for trauma survivors, along with the comfort of being in their own home during therapy sessions:

*‘* *Minimise the time and minimise the journey, minimise everything. Yes, in the end, the fact it brings it down to the comfort of your home, as long as you have the facilities within the home, it’s fine.’*

(Trauma survivor, orthopaedic injury)

Therapists identified two new facilitators. Firstly, they highlighted how remote delivery can improve patient engagement and secondly, they suggested that the remote intervention worked well for trauma survivors able to use technology with reliable internet access:

*‘* *The pros of being able to speak with someone online rather than having to travel is maximising people’s engagement that way. They just have to flick on a computer rather than get ready for a visit and all of that. There’s been no travel time, which is great.’*

(OT05)

Remote delivery of the VR intervention also improved geographical reach, enabling therapists to see trauma survivors from geographical locations which would not have been possible for face-to-face delivery.

##### Beliefs about Capabilities

Therapists identified a new barrier relating to obtaining third-party feedback on trauma survivor progress (e.g., receiving feedback about patient from employer, carer, and colleagues). This would previously have been obtained during workplace or home visits, but reported that this was more difficult to achieve remotely:

*‘* *I was concerned about not getting any objective feedback from people, any third-party feedback…in terms of sort of asking about, trying to be quite clear and saying what feedback are you getting from your employer and asking such questions like, how will you know if things aren’t going quite as well as you think.’*

(OT07)

Concerns about building rapport changed from the pre-intervention interviews; therapists felt they were able to develop therapeutic rapport with trauma survivors, but it took longer than with face-to-face contact, especially where contacts were made by phone not videocall:

*‘* *If you’re purely by phone, it takes a bit longer to build that rapport with somebody than if you’ve seen them and they know who you are’*

(OT03)

They suggested that this could be overcome by conducting an initial face-to-face session, to meet trauma survivors in person and get a better understanding of the environment in which they live, with subsequent sessions delivered remotely:

*‘* *So I would have liked to have met them once face-to-face, initially, I think, for my initial visit. But then I think it would have been—it was perfectly appropriate to carry out the rest of the intervention with my two patients over video.’*

(OT05)

Other new barriers identified included the need to change communication style and problems with internet connectivity. Therapists were more aware of the way they were speaking to trauma survivors if they were unable to see them in person and found it harder to be assertive:

*‘* *And I think for him it’s been challenging to know, just quite how direct to be; and I think it’s possibly harder for that to come across virtually, that you don’t want to come across being like really assertive, and a sort of, well you’re wrong, I’m right type thing.’*

(OT07)

Difficulties connecting to the internet and subsequent inability to conduct functional assessments was frustrating for therapists:

*‘* *If [patient] was not on the ROWTATE study, I’d have been out to the home and gone, “Look, we [therapist and patient] can’t get on virtually. I’ve tried a number of times now. I’m just coming to your house. I want to see your bed mobility. I want to see your transfers. I want to see if there’s more I can get you doing in the kitchen”. We keep wasting chunks of time faffing about with trying to make a connection [online].’*

(OT03)

Both therapists and trauma survivors felt that being able to see someone’s face and gestures, even if it was remotely, was seen as a facilitator to intervention delivery:

*‘* *I think being able to see each other’s faces and gestures [on a video call] was certainly good.’*

(Trauma survivor, polytrauma)

Therapists also felt that video calls were better than phone contact for trauma survivors with communication problems, as they were more able to see if the individual was losing focus or needed extra processing time.

*‘* *So dealing with different levels of communication and cognition, it is easier if, visually, you can see someone. I can tell much more easily if you’re [patient] struggling and need extra processing time if I can see you than if you’re on the phone and you’re just not listening, or whether, actually, you’re [patient] struggling to process what I’m saying. That’s much easier visually. So yeah, I think if you can see them [patient] visually, it’s better.’*

(OT03)

##### Optimism

Therapists felt trauma survivors may perceive online appointments as less important than hospital appointments, as they would need to take time out of their day to visit the hospital, in comparison to a quick video call:

*‘* *There’s something around people having a little bit more, it’s not necessarily respect, but give a little bit more importance perhaps to hospital appointments and face-to-face…there’s been the “could you not do a Saturday”…But then equally you think, well your consultant hasn’t offered you a Saturday appointment, you’d take time off to physically go to see them at hospital. So there’s something about giving it equal merit and weight I think, and an equal importance.’*

(OT07)

Remote delivery may not be suitable for all trauma survivors, or even for all therapists. Some therapists suggested that other therapists may be reluctant to deliver VR remotely and similar to pre-intervention interviews, some trauma survivors preferred face-to-face contact:

*‘* *I think for most things, I actually prefer face-to-face interaction with people, just because I feel that it’s easier to engage with people on a more emotional level as well. It’s also, I think, easier, particularly if you’re very anxious about a situation, to then get support from someone in a meaningful way.’*

(Trauma survivor, polytrauma)

Despite barriers identified by trauma survivors and therapists, all were positive about the remote delivery of the intervention and highlighted its benefits as outlined above. As in the pre-intervention interviews, flexibility was one of the key facilitators of remote delivery, as it enabled sessions to be conducted as and when needed. A new facilitator highlighted by a trauma survivor was that they felt less conscious of their injuries with remote delivery:

*‘* *If I was going to go to a face-to-face meeting, I would’ve been conscious about my injuries, my parking, my walking, and all the other things. In that respect, remotely made life so easy.’*

(Trauma survivor, orthopaedic injury)

##### Social/Professional Role and Identity

Prior to intervention delivery, therapists seemed concerned about changing their normal way of working and adopting novel approaches to conducting therapy sessions. However, post-intervention therapists appeared to be incorporating remote delivery into their professional role and several suggested that they would continue to conduct sessions remotely in the future:

*‘* *Some of those work reviews and stuff that, traditionally, were done face-to-face and would’ve been time consuming. But to do them virtually, because you know the patient really well and you’ve done all the assessments that you need to do face-to-face, it works really well virtually.’*

(OT03)

Therapists also felt that teamworking between occupational therapists and clinical psychologists was an important element of the intervention and had worked well remotely:

*‘* *I think the critical part for me is the contribution that the* *occupational therapists* *are making, in particular in that sort of knitting together…So, communicating and synthesising all of that advice, making those links, how they approach their employer as well, I think the* *occupational therapists* *have really just done a great job in connecting all of that up, and, I guess, connecting them up to psychology as well.’*

(CP06)

##### Acceptability of the Intervention

Findings from post-intervention interviews were mapped onto seven TFA domains (Figure 1) and are summarised below, with supporting quotes and further details on findings given in Table 6.

*Affective attitude:* Both patients and therapists were satisfied with remote delivery despite initial concerns with online mode of delivery.

*Perceived effectiveness:* Both patients and therapists were satisfied with the effectiveness of the intervention delivered which they believed were maintained despite initial challenges of online delivery (e.g., IT issues).

*Burden*: Both patients and therapists could identify the benefits of online delivery in terms of access and time commitment (e.g., minimising travel). A few barriers identified could be overcome with time (e.g., extra time required to familiarise themselves with technology).

*Ethicality*: Online delivery met the shared values for what intervention should be for patients and therapists.

*Opportunity costs*: For all patients and most therapists there were no additional costs for participation in online intervention. All felt the flexibility of the intervention reduced associated costs (e.g., loss of time).

*Intervention coherence*: Both patients and therapists had a good understanding of the value of the intervention and rationale for remote delivery.

*Self-efficacy:* The patients felt the intervention had increased their ability to work on their goals, and therapists overall felt confident in delivery the intervention. 

**Table 6:** Key quotes regarding acceptability obtained from post-intervention therapist and patient interviews.

TFA Construct	Summary of Key Findings	Therapists (Occupational Therapists and Clinical Psychologists)	Patients
Affective attitude	All patients were happy with remote delivery and grateful for the support they had received. All therapists felt the remote delivery of the intervention was acceptable. Many were concerned about building therapeutic rapport online; however, it was not affected for most, but it did take longer than it would face to face.	*Well, I think there’ll be some people who will be really reticent. I think there are therapists who are really reticent to work this way and I’m sure there’ll be some patients for whom the idea is really off-putting and will say, “I’d rather that we do this all face-to-face.” I think in those cases, you would just try and say, “Well, let’s give it a go. We’ll test this out,” and try and orient them to that.’ (CP)* *‘It was actually quite good to do the intervention remotely. It did work in this instance. My participants were both people who are capable of using technology. They had access to the internet. They were appropriately set up to do that.’ (OT)* *‘The feedback from my participant one, from the HR manager was that they thought it was a really positive experience and described it as a luxury to have someone dedicated to supporting their employee back to work’ (OT)* *‘What’s been really good is, across the two sites, to have that sense of we’re all feeling our way through this together, and I think you established that really early on with the original training and that was reinforced with the refresher training’ (CP)* *‘But what has actually been quite positive is that they’ve managed to see my face the whole time and if I was doing face-to-face intervention, you have to obviously be wearing a mask [due to COVID-19].’ (OT)* *‘I think there are therapists who are really reticent to work this way and I’m sure there’ll be some patients for whom the idea is really off-putting and will say, “I’d rather that we do this all face-to-face.”… I’m not sure that people who are that reticent to engage remotely would sign up for an intervention like this in the first place.’ (CP)*	*‘It’s absolutely critical that the occupational therapists have time…I can’t emphasise enough how important it is to give more time to occupational therapists to help patients in this situation.’ (Trauma survivor, TBI)* *‘I wasn’t expecting I was going to go back to work until six months, but because this OT was so adamant and created a strategy, it absolutely made a lot of difference to push me and, before my six months, I was happily doing six hours. I cannot be grateful more than that, I can tell you that, absolutely.’ (Trauma survivor, orthopaedic injury)*
Burden	In some cases, trauma survivors preferred remote delivery to face-to-face sessions as it minimised travel, it was good to stay in the comfort of their home and it meant that sessions could be more flexible. Therapists stated that less time was wasted travelling to appointments and they were able to spend more time delivering rehabilitation. All felt remote delivery had minimal burden, with the only issues being increased workload if the therapist did not specialise in that injury type (e.g., neuro-OT working with orthopaedic trauma survivors) or extra time needed to familiarise themselves with technology.	*‘It’s so much easier because sometimes participants had to cancel at short notice, and rather than, well, we’ll have to wait for the next day that is allocated, we can sometimes pick up the day after, or later that day. You just have that flexibility that if you were travelling between appointments, you just wouldn’t have’ (CP)* *‘It is an effort because, again, adding virtual just makes it something that’s less familiar, I guess, for me. So it is a bit more effortful.’ (OT)* *‘I think it probably was the form filling. Once you’ve got your session up and running, you flow and it’s fine. I’m used to talking to somebody and writing notes, and that’s fine. I think it was the forms and checking have I got it right and how long do I spend on that.’ (CP)*	*“Absolutely great, because whether you see them personally or see them remotely, the way she made a difference in my life within the last few months through remote sessions is immensely high. I mean, if I was going to go to a face-to-face meeting, I would’ve been conscious about my injuries, my parking, my walking, and all the other things. In that respect, remotely made life so easy.” (Trauma survivor, orthopaedic injury)* *So yeah, all things being equal, perhaps in these cases of head injuries at the beginning when you still need a lot of time to sleep so that your body can recover, it may be beneficial, actually, to have contact online (Trauma survivor, TBI)* *‘Minimise the time and minimise the journey, minimise everything. Yes, in the end, the fact it brings it down to the comfort of your home, as long as you have the facilities within the home, it’s fine. But if I had a choice because I have got my facilities, I would say, yes, I prefer a remote session’ (Trauma survivor, orthopaedic injury)*
Ethicality	Most trauma survivors felt the therapist was on the ‘same page’ as them and shared the same values. Some therapists felt that remote intervention delivery fitted well with their new way of working as a result of COVID-19, and how their services would continue to deliver therapy via video or phone call post-pandemic, where possible. Therapists felt that the intervention fitted with their values and enabled them to deliver meaningful rehabilitation like VR, which is often time limited (or not possible) in normal clinical practice.	*Absolutely in line. Yeah, absolutely, because it’s all around quality of life, meaningful activity, participation. Yeah, absolutely in line with what I do normally and what I kind of believe OT should be doing and should be able to look at and should be able to do for people. So yeah, absolutely in line with those values…I’ve really enjoyed the vocational rehab side of things because it’s so what OTs should be doing, in my eyes. It’s so about meaningful activity and positive mood, all those things that come with return to work. So yeah, definitely in line with my OT values. (OT)*	*‘Absolutely, because she was on the same page. As soon as I talk about X, Y and Z, she’s on the same page as what I’m talking about. That made it a lot easier because…I can see it making a difference and it builds my confidence behind it.’ (Trauma survivor, orthopaedic injury)* *‘She was so spot on with my issues and on the same page as I am. That made me more confident behind what she was telling me and just to take it on board at once.’ (Trauma survivor, orthopaedic injury)*
Intervention coherence	All trauma survivors understood the need for VR as they felt they would have returned to work too soon without it. Therapists reported that trauma survivors had been receptive to the remote delivery and understood the purpose of the intervention, acting on therapists’ advice. All therapists understood the purpose of the intervention and knew how to deliver it, stating that training and mentoring was both necessary and helpful.	*‘I developed a good therapeutic relationship with them. I certainly think that they’ve understood the point of the intervention and they’ve both communicated benefiting from the intervention and feeling—particularly one participant saying she was really pleased she’d ticked the box that said, “Yes, I’ll have this intervention.’ (OT)* *‘It is about the case management. It’s about linking in with other people that makes the difference. And from the other perspective, the employers have been mindful and aware of where they are in their journey, so they’ve got that information. Because sometimes, for example, patient three, the one that thought he would get back next week, it could have been a different employer that didn’t really have that understanding and then that expectation would be that he should be back. So, getting in early and just having those communications is good.’ (OT)*	*‘It meant that I was able to understand my accident fully and to negotiate, together with someone who was very knowledgeable about what had happened to me, the best way back for me to fully recover. That meant that also my employer, I think, had huge benefits in terms of getting me back at every stage at the exact level I was able to work properly, but also knowing exactly how much time they ought to give me to recover fully.’ (Trauma survivor, TBI)* *‘That is the right word, strategy. It’s step-by-step building up, and I can feel the difference. Why? Because my energy level wasn’t going down and I was just maintaining myself, happily maintaining my work. I’m doing six hours a day and happily doing it without losing my energy. So, when I come home, I have energy left to do other things as well. And it’s all down to the OT because she put the strategy behind it, and that works.’ (Trauma survivor, orthopaedic injury)* *‘But in these situations, I think it’s all the more important to have someone like [OT] there to help you negotiate, and also to make the employer see that, in the end, it is for their benefit as well because they will get an employee back who is fully employable again and suffers less.’ (Trauma survivor, TBI)*
Opportunity costs	Trauma survivors did not suggest that participating in the intervention had cost them anything in terms of time or money, with all referring to how much time and effort remote delivery had saved. Trauma survivors felt that the flexibility of the remote intervention and ability to rearrange therapy sessions last minute, meant that they did not have to give up anything (e.g., time and plans) to engage in the intervention. Only one therapist mentioned financial costs associated with delivering the intervention, as they decided to pay for an online videoconferencing software to deliver the intervention and had to use their own phone to call trauma survivors.	*So just under £14 a month for my Zoom account. And money, printing costs, printing and paper costs, my phone, I’ve been using my own personal phone, and my own laptop and my own internet’ (OT)* *I think it has cost me time. It has cost me time. As I said, I’ve had to slot the ROWTATE in. Rather than it being a nice extra bit of work for me to do which I’d planned for, I’ve now had to slot it in to this demanding week. (CP)* *What was positive about it is that you can fit in sessions or interventions a lot more easily. They don’t take up as much time because they don’t involve travel. (OT)*	*‘There’s been a few times where I’ve gone, “This is happening and I can’t speak to you then,” and it’s been quite last minute and they’ve gone, “Right, okay, we’ll rearrange it.” So they’ve always been flexible. It’s never cost me in time or money, at all.’ (Trauma survivor, orthopaedic injury)* *‘So I can imagine if you’d have to travel, so where I live it takes me about 55 min to get to the [hospital] and back, so that would be two hours travelling…like that’s not like a small amount of time regardless who you are…so it’s been good being able to have this option rather than have to travel.’ (Trauma survivor, orthopaedic injury)*
Perceived effectiveness	Trauma survivors did not feel online delivery affected the relationship they had with their therapist. Many noticed a difference in their ability to manage their RTW (e.g., taking regular breaks and managing their hours based on fatigue levels) as a result of the intervention and early contact to discuss RTW was important. All therapists felt they were able to deliver the intervention remotely and support their trauma survivor’s RTW. For most therapists, trauma survivors were confident using technology and had access to the internet, therefore were able to engage with the intervention.	*I think it’s gone pretty well in terms of being able to engage people with the remote delivering and building up a rapport and a working relationship. I think that’s gone pretty well and we’ve been able to support people to make some changes remotely.’ (CP)* *‘I feel quite positive about it because the feedback has been quite positive and people have said that they’re really glad they’re taking part in the study and they’re getting this additional support.’ (CP)* *‘I would have liked to have met my clients once face to face. I think it would have given me more idea of the context in which they lived and worked. And I do think it enhances the therapeutic relationship.’ (OT)* *‘It was actually quite good to do the intervention remotely. It did work in this instance. My participants were both people who are capable of using technology. They had access to the internet. They were appropriately set up to do that.’ (OT)*	*‘The work with my occupational therapist, has been invaluable really, and absolutely amazing. [OT) has been very professional, very knowledgeable, and it has made my life so much easier having had that support. It was absolutely brilliant really.’ (Trauma survivor, orthopaedic injury)* *‘I think it’s been brilliant. I think if I didn’t have it, I daren’t think where I’d be now. It’s been really helpful.’ (Trauma survivor, orthopaedic injury)* *‘But then, also, when it came to negotiating with my employer what would be the best return to work for me, she was really invaluable as well. I think both for my employer and for myself, because she really made us understand what would be the best way to make me recovery fully and be back at work, eventually, in a full-time capacity, and sort of not to rush that.’ (Trauma survivor, TBI)*
Self-efficacy	Trauma survivors felt their therapist had helped them to work towards goals and develop insight into the impact of their injury. One issue highlighted by therapists was difficulty gaining consent to speak to employers online, which meant parts of the intervention could not be delivered. Therapists that were initially apprehensive about remote delivery reported that they were now confident conducting sessions via video or phone call.	*‘Think the training was essential. I think it’s really important. Yeah, I think I would’ve tried to use my experience as best as I could for this, but I think I would’ve been kind of flailing a bit, going, oh, hold on a minute.’ (CP)* *‘If there’s anything I’m not sure about from an intervention perspective, I would go back to [OT mentor] and be happy to kind of query that with her. And obviously we have the structured monthly mentoring with her, which has been sufficient in terms of when I’ve needed to ask things so far. But I would equally be happy to contact her if there was intervention stuff I wanted to ask in between times.’ (OT)* *‘I think he’s [patient] been particularly interesting, and a particular challenge, which is good for me and I do like that type of patient and I can thrive on a challenge and I think it’s, you know, if I’m finding things a bit, you know, tricky, I will, you know, obviously you need to alter your approach don’t you depending on the client that you’re working with.’ (OT)* *‘If anything, what it might force, and a big part of that, is promoting self-efficacy. I think when you’re doing it remotely, it really enables one way of thinking about it, because you do feel that much more removed and people know that you’re there and you can offer advice and you’re in contact. I don’t know what you’ll learn from the participants, but the feedback so far, kind of informally, seems somewhat positive about that. But it feels like you’re not alongside them in the same way as maybe you would be normally, and that leaves more to them, I think, or puts more on them.’ (CP)*	*‘I think she’s been perfect. I couldn’t praise her any more, to be honest. She’s been a bit pushy sometimes, but in a good way, to push me to do stuff because, at the start, I wasn’t keen on doing anything, to be honest, because things were taking too long. It’s been, what, six months? It’s nearly seven months now. She’s pushed me and I feel like I’ve overtaken her expectations. So she’s helped me to push myself and now, yeah, I’m really chuffed, honestly.’ (Trauma survivor, orthopaedic injury)* *‘I would also say, having been forced to really think about my work in itemised bits of tasks—although, that was something I was not very happy about when [OT] suggested I do it—was actually very helpful. And, in fact, I told that to a colleague of mine who was saying that she felt quite exhausted from the pandemic and all the home working. I told her about this exercise and she found that quite helpful, too.’ (Trauma survivor, TBI)* *‘It’s an eye-opener regarding how to recover within myself within the working environment because the way I was going was probably the wrong way because I was going straight away into full hours working. She made me realise that if you’re going to have a lack of energy, you’re going to probably not wake up, or not going to come out of bed the next morning because you wear yourself out so quick. So that realisation was so good.’ (Trauma survivor, orthopaedic injury)* *‘I think it changes your thinking about it and you can then be a bit more proactive with like how you do things, where you do things, when you do them, all that sort of stuff.’ (Trauma survivor, TBI)*

OT: occupational therapist; CP: clinical psychologist.

## 4. Discussion

To our knowledge, this study is the first to report on barriers to, facilitators for, and acceptability of a remotely delivered VR intervention for general trauma survivors. It also presents a novel approach to using theory to guide the investigation of facilitators for, and acceptability of telerehabilitation in the context of VR. Overall, the remote VR intervention was acceptable to trauma survivors and therapists across domains of the Theoretical Framework of Acceptability, with the exception of therapists not always being able to engage with employers. By overcoming the accessibility issues that currently limit VR service delivery, our study suggests a new approach to increase access to return-to-work support which could help address service provision inconsistencies across the UK.

Some barriers were common to both pre- and post-intervention interviews: technology and internet access, understanding home or work context, privacy and disruptions, and building therapeutic rapport. The “impossibility” of remote cognitive or workplace assessments, therapists’ lack of knowledge or confidence and professional role or identity were barriers pre-intervention, but not post-intervention. New barriers post-intervention included NHS Trust IT policies, obtaining third-party feedback, lack of importance placed on remote sessions by patients, and patients or other therapists’ reluctance to use remote VR. More facilitators were identified post-intervention than pre-intervention. Facilitators common pre- and post-intervention included reduced travel, increased geographical reach and greater flexibility with service delivery. New facilitators post-intervention included improved patient engagement, positive patient experiences, video calls allowing better communication than phone calls, patients feeling less conscious about their injuries, incorporation of remote working into professional roles and good team working between occupational therapists and CPs.

Our findings regarding technological problems and internet access are consistent with those of other studies [10,15,18,26,32]. Thus, despite increasing access to digital devices and the internet, these issues still remain. They are particularly relevant for patients for whom remote delivery could improve access to rehabilitation (e.g., those living in rural areas at long distances from hospital) or for those with cognitive problems. Unlike other studies, we found that rapidly changing hospital IT policies regarding permitted software platforms were problematic, possibly arising from urgent introduction of remote services during the COVID-19 pandemic and subsequent changes to IT policies. Whilst this may be less of a problem in future, variations in IT policies across hospitals will act as a barrier to multicentre research studies.

Interviewed therapists had concerns that remote delivery could make it difficult to understand the context of patients’ home or work environments, and to get third party feedback on patients’ progress. This is in contrast to a previous study (of a non-VR intervention) which found that video calls helped therapists obtain “collateral” information about patient functioning, family interaction, and the home environment [38]. This difference may reflect the need to understand the work environment and functioning at work as part of our VR intervention. Our pre-intervention findings relating to privacy and disruptions are also consistent with those of other studies [38,39], including therapists’ concerns regarding communication with patients and building rapport [31,32,39,40,41].

Post-intervention interviews suggested that receiving training in remote delivery and experience of delivering VR remotely was necessary for developing confidence to use telerehabilitation modalities. Similar to other studies, therapists who had used video calls to deliver VR realised they were able to build good rapport with patients [38], consistent with studies which found that clinicians’ acceptance of remote intervention delivery was moderated by experience [10,42], with hands-on experience being a key facilitator to implementation [40,42]. Despite the benefits of remote delivery, consistent with other studies, we found that some face-to-face delivery may still be required [38,40,42], such as conducting risk assessments (especially for brain injury survivors), visiting the workplace of some non-office-based roles and an initial meeting to ensure trauma survivors are able to join future sessions online (e.g., setting up equipment). We did not find therapists’ concerns regarding communication were echoed by patients. Previous studies report conflicting findings; some found that telerehabilitation patients report positive relationships with providers [39], whilst others report patients may feel less comfortable with remote delivery [41]. Our findings regarding therapists perceiving patients placing less importance on remote than face-to-face therapy sessions has not been found in other studies.

Several key facilitators were identified corroborating previous studies, including reduced travel time [10,12,13,15,38,39,40], improved access and geographical reach [10,15,38,39,40,41], positive patient experiences of telerehabilitation [10,15,39,41,42,43,44], increased flexibility in service delivery associated with remote delivery [10,38,40,42,44] and enhanced communication and teamworking between health care providers [15]. Patients felt that receiving remote therapy in the comfort of their own home helped them to engage in sessions, for example, because they felt less conscious about injuries than in a face-to-face meeting. Other studies have also reported that patients preferred remote delivery because they felt more comfortable at home [44], liked the anonymity it afforded or because it reduced stigma, avoided triggers for post-traumatic stress disorder or avoided travel-related anxiety [39]. To our knowledge, our finding that therapists who had received training and used remote delivery started to incorporate it into their professional role or identity has not been reported by other studies.

Our study had several strengths. Firstly, it analysed the perspectives of both patients and therapists to identify similar and divergent barriers and facilitators. Data analysis was enriched by involving authors with lived experience of trauma in the interpretation of findings. Secondly, we were able to identify how barriers and facilitators changed with experience of delivering telerehabilitation. Thirdly, we used different methodologies which have different strengths in terms of the data they provide, and both these methodologies were informed by a multidisciplinary team of researchers, practitioners and trauma patients. However, our sample size was small, preventing statistical comparison of changes in behavioural determinants pre- and post-training. It is also possible that non-responders to the post-training survey may have held more negative views than responders and our post-training survey may have failed to identify some barriers. However, we also interviewed therapists post-intervention and two of the three not returning the post-training survey were interviewed, so their views are included in the qualitative analysis. It is possible that previous contact between interviewers and interviewees or interviewees’ knowledge that the research was aimed at developing the intervention for a definitive trial may have influenced data collection. Interviewers had a good rapport with interviewees, enabling interviewees to be put at ease during the interviews, which may have encouraged honesty about their experiences, or may have inhibited expression of negative views. However, the range of positive and negative views we elicited suggests that interviewees did feel able to express a variety of views. Only 40% of patients recruited to the feasibility study were interviewed, and again, those not interviewed may have held differing views to those interviewed. The main limitation to our study was failure to interview employers, which resulted from patients being self-employed, patients not consenting for researchers to contact employers and employers not responding to study invites. The views of employers regarding remote delivery of our intervention are therefore unknown.

### Implications for Research and Practice

Developing or adapting VR interventions for remote delivery is going to become increasingly important as services move from traditional face-to-face delivery models. Our findings provide insight into the barriers which need to be addressed in the design of remotely delivered VR interventions, or the adaption of existing VR interventions for remote delivery. These include a range of IT barriers for patients and therapists, allowing flexibility so that face-to-face delivery can be provided where necessary and strategies to enhance privacy, minimise disruptions and increase employer engagement. Larger studies exploring acceptability and barriers to and facilitators for delivery, including the views of employers, are required to inform future intervention development.

## Figures and Tables

**Table 1:** Feasibility study objectives mapped onto study methods and description of how the findings have been used to inform the definitive trial.

Feasibility Study Objectives	Methods to Address Objectives
1. Adapt the ROWTATE intervention to make it suitable for remote delivery, as much as possible, via telerehabilitation and tele-psychology	Review of telerehabilitation literature. *Informed intervention development.* Pre-training TDF survey completed by occupational therapists and clinical psychologists to identify determinants of health professional behaviour that may influence intervention delivery. *Informed intervention development and delivery.* Pre-training semi-structured interview with occupational therapists and clinical psychologist to explore current use, barriers, and options for remote delivery. *Informed intervention development and delivery.*
2. Adapt the ROWTATE occupational therapist and clinical psychologist training to make it suitable for remote delivery	Pre-training TDF survey completed by occupational therapists and clinical psychologists to identify determinants of health professional behaviour that may influence intervention delivery. *Informed adaptation of remote intervention delivery therapist training.* Pre-intervention interview with occupational therapists and clinical psychologist to explore current use, barriers, and options for remote delivery. *Informed adaptation of remote intervention delivery therapist training, ensuring specific issues and barriers were addressed during training sessions.*
3. Assess acceptability, barriers and facilitators to remote delivery of the ROWTATE intervention via telerehabilitation and tele-psychology	Post-training TDF survey completed by occupational therapists and clinical psychologists to identify determinants of health professional behaviour that may influence intervention delivery. *Identified barriers/facilitators to remote delivery of the intervention and highlighted any remaining issues that had not been addressed in training. Outstanding issues informed intervention development and delivery for the definitive trial.* Post-intervention interview with trauma survivors receiving the intervention and occupational therapists and clinical psychologists delivering the intervention and employers of recruited patients. Interviews explored barriers and facilitators to the remote delivery of the intervention, along with the acceptability of the intervention. *Issues arising informed intervention development and delivery for the definitive trial.*

**Table 2:** Interview coding framework.

Framework	Code	Description
Theoretical Framework of Acceptability (TFA)	Affective attitude	How an individual feels about an intervention
	Burden	The perceived amount of effort that is required to deliver or participate in the intervention
	Ethicality	The extent to which the intervention has good fit with an individual’s value system
	Intervention coherence	The extent to which the therapist or participant understands the intervention and how it works
	Opportunity costs	The extent to which benefits, profits or values must be given up to deliver or engage in the intervention
	Perceived effectiveness	The extent to which the intervention is perceived as likely to achieve its purpose
	Self-efficacy	The therapist’s or participant’s confidence that they can perform the behaviour(s) required to participate in the intervention
Theoretical Domains Framework (TDF)	Behavioural regulation	Anything aimed at managing or changing objectively observed or measured actions
	Beliefs about capabilities	Acceptance of the truth, reality, or validity about an ability, talent, or facility that a person can put to constructive use
	Beliefs about consequences	Acceptance of the truth, reality, or validity about outcomes of a behaviour in a given situation
	Emotions	A complex reaction pattern, involving experiential, behavioural, and physiological elements, by which the individual attempts to deal with a personally significant matter or event
	Environmental context and resources	Any circumstance of a person’s situation or environment that discourages or encourages the development of skills and abilities, independence, social competence, and adaptive behaviour
	Goals	Mental representation of outcomes or end states that an individual wants to achieve
	Intentions	A conscious decision to perform a behaviour or a resolve to act in a certain way
	Knowledge	An awareness of the existence of something
	Memory, attention and decision processes	The ability to retain information, focus selectively on aspects of the environment, and choose between two or more alternatives
	Optimism	The confidence that things will happen for the best, or that desired goals will be attained
	Reinforcement	Increasing the probability of a response by arranging a dependent relationship, or contingency, between the response and a given stimulus
	Skills	An ability or proficiency acquired through practice
	Social influences	Those interpersonal processes that can cause an individual to change their thoughts, feelings, or behaviours
	Social-professional role and identity	A coherent set of behaviours and displayed personal qualities of an individual in a social or work setting

**Table 3:** Summary of therapist participant characteristics (n = 7).

						TDF Survey		Interview	
Participant ID	Profession	Years Experience	Specialist Area	VR Experience	Telerehab Experience	Pre-Training	Post-Training	Pre-Intervention	Post-Intervention
OT01	OT	20+	TBI, SCI						
CP02	CP	15–19	Stroke						
OT03	OT	15–19	TBI						
OT04	OT	5–9	Trauma						
OT05	OT	20+	TBI						
CP06	CP	5–9	ABI, neurological conditions						
OT07	OT	15–19	TBI						

ABI: acquired brain injury; CP: clinical psychologist; OT: occupational therapist; SCI: spinal cord injury; TBI: traumatic brain injury; TDF: theoretical domains framework; VR: vocational rehabilitation.

**Table 4:** Summary of trauma patient participant characteristics (n = 4).

Participant ID	Age	Sex	ISS Score	Injury Type	Professional Industry	Size of Employing Organisation (Number of Employees)	Consent to Interview Employer?
P01	51	Female	16	Polytrauma including TBI	Curator	250+	Yes *Contact made but no response*
P02	54	Male	9	Orthopaedic	Gas engineer	Self-employed	NA *Self-employed*
P03	33	Male	9	Head and neck injury, TBI	Recruitment consultant	50–249	No
P04	41	Male	9	Orthopaedic	Lorry driver	0–9	No

ISS: injury severity score; NA: not applicable; TBI: traumatic brain injury.

**Table 5:** Pre- and post-training TDF survey data.

		Pre-Training (n = 7)		Post-Training (n = 4)	
TDF Domain	Survey Statements	TDF Domain Mean (SD)	Question Mean (SD)	TDF Domain Mean (SD)	Question Mean (SD)
Intentions	1. I intend to apply telerehabilitation protocols to each/every one of my patients’ sessions	3.4	3.50 (0.50)	5.42	5.50 (1.29)
	2. I will definitely apply telerehabilitation protocols to each/every one of my patients’ sessions	−0.23	3.14 (0.69)	−0.14	5.25 (1.50)
	3. I have a strong intention to apply telerehabilitation protocols to each/every one of my patients’ sessions		3.57 (0.53)		5.50 (1.29)
Social influences	1. People who are important to me think that I should deliver therapy using telerehabilitation	5	4.29 (1.50)	6	5.25 (0.96)
	2. People whose opinion I value would approve of me delivering therapy using telerehabilitation	−0.55	4.86 (0.90)	−0.5	6.25 (0.50)
	3. I can count on support from colleagues whom I work with when things get tough with delivering therapy sessions using telerehabilitation		5.43 (0.79)		6.25 (0.50)
	4. Colleagues whom I work with are willing to listen to the problems I have when delivering therapy sessions using telerehabilitation		5.43 (0.79)		6.25 (0.50)
Knowledge	1. I am aware of the content of an effective telerehabilitation programme	5.03	4.57 (1.27)	6.1	6.25 (0.50)
	2. I am aware of the objectives of a telerehabilitation programme	−0.34	5.00 (0.58)	−0.22	6.25 (0.50)
	3. I know what my responsibilities are, with regard to delivering a therapy session using telerehabilitation		5.29 (0.49)		6.25 (0.50)
	4. I know how to use telerehabilitation		4.86 (0.69)		5.75 (0.50)
	5. I know when to use telerehabilitation		5.43 (0.53)		6.00 (0.82)
	1. I have received training regarding how to deliver telerehabilitation	3.76	1.71 (0.76)	5.92	6.00 (0.82)
Skills	2. I have the skills needed to deliver a telerehabilitation programme	−1.78	4.86 (0.69)	−0.38	5.50 (0.58)
	3. I have been able to practice using telerehabilitation		4.71 (1.25)		6.25 (0.50)
Memory, attention, and decision processes	1. Using telerehabilitation to deliver each of my patients’ sessions is something I do automatically	3.57	3.57 (0.79)	4.75	4.75 (0.96)
		0		−0.96	
Social/professional role and identity	1. Delivering therapy sessions using telerehabilitation is part of my role	4.52	4.86 (0.69)	6.33	6.75 (0.50)
	2. It is my responsibility to delivery therapy sessions using telerehabilitation protocols	−0.08	4.71 (1.25)	−0.38	6.25 (0.50)
	3. Delivering therapy sessions using telerehabilitation is consistent with other aspects of my job		4.43 (1.27)		6.00 (1.41)
Beliefs about capabilities	1. I am confident that I can plan and deliver therapy sessions with my patients using telerehabilitation protocols	4.81	5.14 (0.90)	5.54	6.00 (0.82)
	2. I am capable of planning and delivering telerehabilitation, even when little time is available	−0.45	5.00 (0.82)	−0.62	5.50 (1.00)
	3. I have the confidence to plan and deliver therapy using telerehabilitation even when other professionals I work with are not doing this		5.14 (0.90)		5.75 (0.50)
	4. I have the confidence to plan and deliver therapy using telerehabilitation even when the patients who attend the service are not receptive		4.57 (0.53)		5.25 (0.50)
	5. I have personal control over planning and delivering therapy using telerehabilitation		5.00 (1.00)		6.25 (0.50)
	6. For me, planning and delivering therapy using telerehabilitation is easy		4.00 (0.58)		4.50 (1.00)
Optimism	1. In uncertain times, when I plan and deliver therapy using telerehabilitation I usually expect that things will work out okay	4.05	4.29 (0.76)	5.08	5.50 (0.58)
	2. When I plan and deliver therapy using telerehabilitation, I feel optimistic about my job in the future	−0.3	4.14 (0.90)	−0.72	5.50 (0.58)
	3. I do not expect anything will prevent me from using telerehabilitation to deliver therapy to my patients		3.71 (1.60)		4.25 (0.96)
Beliefs about consequences	1. I believe delivering each of my patients’ sessions using telerehabilitation will lead to benefits for the patients who attend the service	4.55	4.57 (1.27)	5.75	5.75 (0.50)
	2. I believe delivering each of my patients’ sessions using telerehabilitation will benefit public health (i.e., health of the whole population)	−0.04	4.57 (0.79)	−0.41	5.25 (0.96)
	3. In my view, using telerehabilitation to deliver each of my patients’ sessions is useful		4.50 (0.76)		5.75 (0.50)
	4. In my view, using telerehabilitation to deliver each of my patients’ sessions is worthwhile		4.57 (0.79)		6.25 (0.50)
Reinforcement	1. I get recognition from management at the organisation where I work, when I use telerehabilitation to deliver my patients’ sessions	4.38	4.13 (0.38)	5.58	5.50 (1.00)
	2. When I use telerehabilitation to deliver my patients’ sessions, I get recognition from my colleagues	−0.22	4.43 (0.98)	−0.38	5.25 (0.98)
	3. When I use telerehabilitation to deliver my patients’ sessions, I get recognition from those whom it impacts		4.57 (1.13)		6.00 (0.00)
Environmental context and resources	1. In the organisation I work, all necessary resources are available to allow me to deliver my planned therapy using telerehabilitation	3.79	2.43 (1.27)	5.75	5.25 (0.50)
	2. I have support from the management of the organisation to deliver my planned therapy using telerehabilitation	−0.91	4.29 (1.60)	−0.47	6.25 (0.96)
	3. The management of the organisation I work for are willing to listen to any problems I have when delivering my planned therapy using telerehabilitation		4.86 (1.22)		6.25 (0.50)
	4. The organisation I work for provides the opportunity for training to deliver my planned therapy using telerehabilitation		3.50 (0.96)		5.50 (1.73)
	5. The organisation I work for provides sufficient time for me to deliver my planned therapy using telerehabilitation		3.86 (1.46)		5.50 (0.58)
Goals	1. Compared to my other tasks, planning how and delivering my therapy using telerehabilitation is a higher priority on my agenda	4.76	4.86 (0.69)	5	4.25 (1.71)
	2. Compared to my other tasks, planning how and delivering my therapy using telerehabilitation is an urgent item on my agenda	−0.3	4.43 (0.53)	−0.9	4.75 (0.96)
	3. I have clear goals related to using telerehabilitation to deliver each of my patients’ sessions		5.00 (1.00)		6.00 (0.00)
Behavioural regulation	1. I have a detailed plan of how I will deliver therapy using telerehabilitation	4.37	4.14 (0.69)	5.3	5.50 (0.58)
	2. I have a detailed plan of how I will deliver therapy using telerehabilitation when patients who usually attend the service are not receptive	−0.47	3.86 (0.69)	−0.41	4.75 (0.50)
	3. I have a detailed plan of how I will deliver therapy using telerehabilitation when there is little time		4.14 (0.69)		5.00 (0.82)
	4. It is possible to adapt how I will deliver therapy using telerehabilitation to meet my needs as a rehabilitation therapist/psychologist		4.71 (0.76)		5.50 (0.58)
	5. Delivering therapy using telerehabilitation is compatible with other aspects of my job		5.00 (0.93)		5.75 (0.96)
Emotion	1. I am able to deliver therapy using telerehabilitation without feeling anxious	4.91	4.14 (1.22)	5.58	5.50 (0.58)
	2. I am able to deliver therapy using telerehabilitation without feeling distressed or upset	−0.66	5.29 (0.76)	−0.14	5.75 (0.50)
	3. I am able to deliver therapy using telerehabilitation, even when I feel stressed		5.29 (0.49)		5.50 (1.00)

Red highlighted mean domain scores indicate substantial barriers (scores ≤ 3.5) and green highlighted mean domain scores indicate enabling facilitators (scores ≥ 5) to implementing the remote VR intervention. SD: standard deviation; TDF: theoretical domains framework.

## Data Availability

The data that participants have consented to share will become available to potential researchers at the end of this study. Requests detailing the research aims and use of the data should be sent to the research team via email: ROWTATE@nottingham.ac.uk.

## Supplementary Materials

The following are available online at https://www.mdpi.com/article/10.3390/ijerph18189744/s1, Survey S1: ROWTATE pre-training therapist telerehabilitation questionnaire, Survey S2: ROWTATE post-training therapist telerehabilitation questionnaire, Topic Guide S3: therapists pre-intervention interview topic guide, Topic Guide S4: patient post-intervention interview topic guide, Topic Guide S5: therapists post-intervention interview topic guide.
