# Supplementary material for: Factors Affecting the Delivery and Acceptability of the ROWTATE Telehealth Vocational Rehabilitation Intervention for Traumatic Injury Survivors: A Mixed-Methods Study
Source: Int J Environ Res Public Health. 2021 Sep 16;18(18):9744. doi: 10.3390/ijerph18189744 (PMC8471954; doi:10.3390/ijerph18189744)
Supplement: Supplementary file 1 [file ijerph-18-09744-s001.zip › ijerph-1365000-supplementary.pdf]

Supplementary File 1

ROWTATE Therapist Telerehabilitation Questionnaire

Page 1: Welcome

Thank you for agreeing to take part in this questionnaire.

We want to find out more about your current practices and whether they have changed as a result of COVID19. We are particularly interested in your experiences of using telerehabilitation and your opinions of delivering therapy sessions in this way.

Your feedback will help us to adapt the ROWTATE intervention so that it can be delivered during the current pandemic.

Any names or services you mention in your answers will remain confidential and will be anonymised.

Page 2: A little about you

What is your main health profession?

- ○ Occupational therapist
- ○ Clinical psychologist
- ○ Other

If you selected Other, please specify:

How many years experience do you have in this role?

- ○ Less than 1 year
- ○ 1-4 years
- ○ 5-9 years
- ○ 10-14 years
- ○ 15-19 years
- ○ 20 years or more

Please briefly describe your usual clinical caseload: ✱ *Required*

How many days a week do you see patients clinically? ✱ *Required*

How many patients do you see in an average day? ✱ *Required*

What is your specialist area?

- ☐ Stroke
- ☐ Traumatic brain injuries
- ☐ Musculoskeletal injuries
- ☐ Spinal cord injuries
- ☐ Amputees
- ☐ Other (please describe)

If you selected Other, please specify:

Do you have experience of delivering vocational rehabilitation? ✱ *Required*

- ◯ Yes
- ◯ No

If you do have experience of delivering vocational rehabilitation, please provide more details (including patient type, years of experience, method of delivery)?

Page 3: Using technology

For each of the following statements about the use of computers / devices / technology in healthcare, please indicate if you strongly agree, somewhat agree, somewhat disagree, or strongly disagree: ✱ *Required*

	Strongly Agree	Somewhat Agree	Somewhat Disagree	Strongly Disagree
It is important that I use the most up to date technology systems in my practice	☐	☐	☐	☐
In my sector, it is expected that I use technology as part of my daily work	☐	☐	☐	☐
Most practitioners in my professional network use technology regularly within their practice	☐	☐	☐	☐
Using technology helps reduce the risk of error in my sector	☐	☐	☐	☐
I would like computerised access to a shared patient summary that includes basic health records	☐	☐	☐	☐
I like to be an early adopter of new technology systems and software for my practice	☐	☐	☐	☐

This is a question about 'telerehabilitation' which is the use of broadband-enabled information and communication technology (including telephones) to deliver health services, expertise and information remotely. It includes both clinical elements of the health care system such as remote consultations with patients, and non-clinical elements such as remote training. By telerehabiliation, we are referring to the delivery of rehabilitation or psychological support. **Are you already using any telerehabilitation services?**

- ○ Yes
- ○ No

Which of the following video call/telerehabilitation platforms does your NHS Trust **currently allow** you to use?

- ☐ Zoom
- ☐ Skype
- ☐ accuRx
- ☐ FaceTime
- ☐ Microsoft Teams
- ☐ WhatsApp
- ☐ Other

If you selected Other, please specify:

Page 4: Impact of COVID19

Before COVID-19, at which of the following locations did you see patients for rehabilitation? Tick all that apply

- ☐ At a hospital
- ☐ At a clinic/outpatient practice location (not attached to a hospital)
- ☐ At a patient's home or premises
- ☐ At a community health centre
- ☐ In the community
- ☐ Using telemedicine/online methods (includes telephone appointments, video-conferencing, telerehabilitation etc)
- ☐ Other
- ☐ Other

If you selected Other, please specify:

Please indicate whether you had previously used telerehabilitation services to deliver any of the following **before COVID19**:

Please don't select more than 1 answer(s) per row.

	Yes	No
Supervising/monitoring patient therapy sessions remotely	☐	☐
Assessing patients	☐	☐
Holding consultations with patients	☐	☐
Holding consultations with other healthcare practitioners	☐	☐
Delivering a therapy session	☐	☐
Training carers	☐	☐
Training other healthcare practitioners	☐	☐
Supervising other healthcare practitioners	☐	☐

Group programmes/therapy sessions (e.g. exercise groups)	☐	☐
Conducting cognitive assessments	☐	☐
Delivering psychological interventions	☐	☐

If you have previously used telerehabilitation for other activities, please specify:

Please indicate whether you had previously used each of the following technologies to deliver telerehabilitation **before COVID19**:

Please don't select more than 1 answer(s) per row.

	Yes	No
Specialist online consultation systems (such as accuRx)	☐	☐
Videoconferencing (such as Zoom, Skype, Microsoft Teams)	☐	☐
Smartphone calls using video (such as Facetime)	☐	☐
Telephone calls	☐	☐
Text messaging	☐	☐
WhatsApp messaging	☐	☐
Activity monitors (such as Fit-bit) for remote monitoring of activity levels	☐	☐

Please indicate whether you had previously used telerehabilitation services to deliver any of the following **as a result of COVID19**:

Please don't select more than 1 answer(s) per row.

	Yes	No
Supervising/monitoring patient therapy sessions remotely	☐	☐
Assessing patients	☐	☐
Holding consultations with patients	☐	☐

Holding consultations with other healthcare practitioners	☐	☐
Delivering a therapy session	☐	☐
Training carers	☐	☐
Training other healthcare practitioners	☐	☐
Supervising other healthcare practitioners	☐	☐
Group programmes/therapy sessions (e.g. exercise groups)	☐	☐
Conducting cognitive assessments	☐	☐
Delivering psychological interventions	☐	☐

Please indicate whether you have used each of the following technologies to deliver telerehabilitation **as a result** of COVID19

Please don't select more than 1 answer(s) per row.

	Yes	No
Specialist online consultation systems (such as accuRx)	☐	☐
Videoconferencing (such as Zoom, Skype, Microsoft Teams)	☐	☐
Smartphone calls using video (such as Facetime)	☐	☐
Telephone calls	☐	☐
Text messaging	☐	☐
WhatsApp messaging	☐	☐
Activity monitors (such as Fit-bit) for remote monitoring of activity levels	☐	☐

Page 5: Delivering telerehabilitation

We would like to understand more about how you currently feel about telerehabilitation (including delivery of rehabilitation and psychological support). There are no right or wrong answers. Please select the response that relates most closely to your current situation and experience.

Please don't select more than 1 answer(s) per row.

	Strongly Agree	Agree	Somewhat Agree	Neither Agree not Disagree	Somewhat Disagree	Disagree	Strongly Disagree
I am aware of the content of an effective telerehabilitation programme	☐	☐	☐	☐	☐	☐	☐
I am aware of the objectives of a telerehabilitation programme	☐	☐	☐	☐	☐	☐	☐
I know what my responsibilities are, with regard to delivering a therapy session using telerehabilitation	☐	☐	☐	☐	☐	☐	☐
I know how to use telerehabilitation	☐	☐	☐	☐	☐	☐	☐
I know when to use telerehabilitation	☐	☐	☐	☐	☐	☐	☐
I have received training regarding how to deliver telerehabilitation	☐	☐	☐	☐	☐	☐	☐

I have the skills needed to deliver a telerehabilitation programme	☐	☐	☐	☐	☐	☐	☐
I have been able to practice using telerehabilitation	☐	☐	☐	☐	☐	☐	☐
Delivering therapy sessions using telerehabilitation is part of my role	☐	☐	☐	☐	☐	☐	☐
It is my responsibility to delivery therapy sessions using telerehabilitation protocols	☐	☐	☐	☐	☐	☐	☐
Delivering therapy sessions using telerehabilitation is consistent with other aspects of my job	☐	☐	☐	☐	☐	☐	☐

We would like to understand more about your confidence of delivering telerehabilitation and whether you feel able to deliver rehabilitation and/or psychological support in this way. Please select the response that relates most closely to your current situation and experience.

Please don't select more than 1 answer(s) per row.

	Strongly Agree	Agree	Somewhat Agree	Neither Agree not Disagree	Somewhat Disagree	Disagree	Strongly Disagree

I am confident that I can plan and deliver therapy sessions with my patients using telerehabilitation protocols	☐	☐	☐	☐	☐	☐	☐
I am capable of planning and delivering telerehabilitation, even when little time is available	☐	☐	☐	☐	☐	☐	☐
I have the confidence to plan and delivery therapy using telerehabilitation even when other professionals I work with are not doing this	☐	☐	☐	☐	☐	☐	☐
I have the confidence to plan and deliver therapy using telerehabilitation even when the patients who attend the service are not receptive	☐	☐	☐	☐	☐	☐	☐
I have personal control over planning and delivering therapy using telerehabilitation	☐	☐	☐	☐	☐	☐	☐

For me, planning and delivering therapy using telerehabilitation is easy	☐	☐	☐	☐	☐	☐	☐
In uncertain times, when I plan and deliver therapy using telerehabilitation I usually expect that things will work out okay	☐	☐	☐	☐	☐	☐	☐
When I plan and deliver therapy using telerehabilitation, I feel optimistic about my job in the future	☐	☐	☐	☐	☐	☐	☐
I do not expect anything will prevent me from using telerehabilitation to deliver therapy to my patients	☐	☐	☐	☐	☐	☐	☐

We would like to understand more about your beliefs and motivations for using telerehabilitation (including delivery of rehabilitation and psychological support). *There are no right or wrong answers. Please select the response that relates most closely to your current situation and experience.*

Please don't select more than 1 answer(s) per row.

	Strongly Agree	Agree	Somewhat Agree	Neither Agree not Disagree	Somewhat Disagree	Disagree	Strongly Disagree

I believe delivering each of my patients' sessions using telerehabilitation will lead to benefits for the patients who attend the service	☐	☐	☐	☐	☐	☐	☐
I believe delivering each of my patients' sessions using telerehabilitation will benefit public health (ie. health of the whole population)	☐	☐	☐	☐	☐	☐	☐
In my view, using telerehabilitation to deliver each of my patients' sessions is useful	☐	☐	☐	☐	☐	☐	☐
In my view, using telerehabilitation to deliver each of my patients' sessions is worthwhile	☐	☐	☐	☐	☐	☐	☐

I get recognition from management at the organisation where I work, when I use telerehabilitation to deliver my patients' sessions	☐	☐	☐	☐	☐	☐	☐
When I use telerehabilitation to deliver my patients' sessions, I get recognition from my colleagues	☐	☐	☐	☐	☐	☐	☐
When I use telerehabilitation to deliver my patients' sessions, I get recognition from those whom it impacts	☐	☐	☐	☐	☐	☐	☐
I intend to apply telerehabilitation protocols to each/every one of my patients' sessions	☐	☐	☐	☐	☐	☐	☐
I will definitely apply telerehabilitation protocols to each/every one of my patients' sessions	☐	☐	☐	☐	☐	☐	☐

I have a strong intention to apply telerehabilitation protocols to each/every one of my patients' sessions	☐	☐	☐	☐	☐	☐	☐

We would like to understand more about how you currently feel about telerehabilitation and the support you receive to deliver rehabilitation in this way. *There are no right or wrong answers. Please select the response that relates most closely to your current situation and experience.*

Please don't select more than 1 answer(s) per row.

	Strongly Agree	Agree	Somewhat Agree	Neither Agree nor Disagree	Somewhat Disagree	Disagree	Strongly Disagree
Compared to my other tasks, planning how and delivering my therapy using telerehabilitation is a higher priority on my agenda	☐	☐	☐	☐	☐	☐	☐
Compared to my other tasks, planning how and delivering my therapy using telerehabilitation is an urgent item on my agenda	☐	☐	☐	☐	☐	☐	☐

I have clear goals related to using telerehabilitation to deliver each of my patients' sessions	☐	☐	☐	☐	☐	☐	☐
Using telerehabilitation to deliver each of my patients' sessions is something I do automatically	☐	☐	☐	☐	☐	☐	☐
In the organisation I work, all necessary resources are available to allow me to deliver my planned therapy using telerehabilitation	☐	☐	☐	☐	☐	☐	☐
I have support from the management of the organisation to deliver my planned therapy using telerehabilitation	☐	☐	☐	☐	☐	☐	☐

The management of the organisation I work for are willing to listen to any problems I have when delivering my planned therapy using telerehabilitation	☐	☐	☐	☐	☐	☐	☐
The organisation I work for provides the opportunity for training to deliver my planned therapy using telerehabilitation	☐	☐	☐	☐	☐	☐	☐
The organisation I work for provides sufficient time for me to deliver my planned therapy using telerehabilitation	☐	☐	☐	☐	☐	☐	☐
People who are important to me think that I should deliver therapy using telerehabilitation	☐	☐	☐	☐	☐	☐	☐
People whose opinion I value would approve of me delivering therapy using telerehabilitation	☐	☐	☐	☐	☐	☐	☐

I can count on support from colleagues whom I work with when things get tough with delivering therapy sessions using telerehabilitation	☐	☐	☐	☐	☐	☐	☐
Colleagues whom I work with are willing to listen to the problems I have when delivering therapy sessions using telerehabilitation	☐	☐	☐	☐	☐	☐	☐

We would like to understand more about how you currently feel about telerehabilitation. *There are no right or wrong answers. Please select the response that relates most closely to your current situation and experience.*

Please don't select more than 1 answer(s) per row.

	Strongly Agree	Agree	Somewhat Agree	Neither Agree nor Disagree	Somewhat Disagree	Disagree	Strongly Disagree
I am able to deliver therapy using telerehabilitation without feeling anxious	☐	☐	☐	☐	☐	☐	☐
I am able to deliver therapy using telerehabilitation without feeling distressed or upset	☐	☐	☐	☐	☐	☐	☐

I am able to deliver therapy using telerehabilitation, even when I feel stressed	☐	☐	☐	☐	☐	☐	☐
I have a detailed plan of how I will deliver therapy using telerehabilitation	☐	☐	☐	☐	☐	☐	☐
I have a detailed plan of how I will deliver therapy using telerehabilitation when patients who usually attend the service are not receptive	☐	☐	☐	☐	☐	☐	☐
I have a detailed plan of how I will deliver therapy using telerehabilitation when there is little time	☐	☐	☐	☐	☐	☐	☐
It is possible to adapt how I will deliver therapy using telerehabilitation to meet my needs as a rehabilitation therapist/psychologist	☐	☐	☐	☐	☐	☐	☐
Delivering therapy using telerehabilitation is compatible with other aspects of my job	☐	☐	☐	☐	☐	☐	☐

Page 6: Improving telerehabilitation

We would like to know more about your experiences of delivering rehabiltiation and/or psychological support **as a result of COVID19**. Please could you tell us about any **barriers or issues** within your NHS Trust that **affect the delivery** of telerehabilitation? *Your responses will remain confidential.*

Is there anything that could **facilitate** (or has facilitated) the delivery of telerehabilitation in your NHS Trust **during COVID19**? (e.g. equipment loans, change of policies). Please tell us how you think things could be improved. *Your responses will remain confidential.*

Page 7: Final page

Thank you for taking part in this study.

We appreciate you taking the time to complete the questionnaire. Your feedback and experiences will help us adapt the ROWTATE intervention to be delivered via telerehabilitation and improve virtual rehabilitation services in the NHS, during COVID19 and in the future.

http://www.ROWTATE.org.uk

Supplementary File 2

ROWTATE TDF survey post-training

Page 1: Welcome

Thank you for agreeing to take part in this questionnaire.

We want to find out more about your current practices and whether they have changed following the ROWTATE telerehabilitation training.

Your feedback will help us to adapt the ROWTATE intervention and training ahead of the definitive trial.

Your answers will remain confidential and will be anonymised.

Page 2: A little about you

Please provide your initials.

What is your main health profession?

- Occupational therapist
- Clinical psychologist

Page 3: Using technology

For each of the following statements about the use of computers / devices / technology in healthcare, please indicate if you strongly agree, somewhat agree, somewhat disagree, or strongly disagree: ✱ *Required*

	Strongly Agree	Somewhat Agree	Somewhat Disagree	Strongly Disagree
It is important that I use the most up to date technology systems in my practice	☐	☐	☐	☐
In my sector, it is expected that I use technology as part of my daily work	☐	☐	☐	☐
Most practitioners in my professional network use technology regularly within their practice	☐	☐	☐	☐
Using technology helps reduce the risk of error in my sector	☐	☐	☐	☐
I would like computerised access to a shared patient summary that includes basic health records	☐	☐	☐	☐
I like to be an early adopter of new technology systems and software for my practice	☐	☐	☐	☐

Which of the following video call/telerehabilitation platforms does your NHS Trust **currently allow** you to use?

- ☐ Zoom
- ☐ Skype
- ☐ accuRx
- ☐ FaceTime
- ☐ Microsoft Teams
- ☐ WhatsApp
- ☐ Other

If you selected Other, please specify:

Page 4: Impact of COVID19

Please indicate whether you have recently used any of the following technologies to deliver telerehabilitation.

Please don't select more than 1 answer(s) per row.

	Yes	No
Specialist online consultation systems (such as accuRx)	☐	☐
Videoconferencing (such as Zoom, Skype, Microsoft Teams)	☐	☐
Smartphone calls using video (such as Facetime)	☐	☐
Telephone calls	☐	☐
Text messaging	☐	☐
WhatsApp messaging	☐	☐
Activity monitors (such as Fit-bit) for remote monitoring of activity levels	☐	☐

Page 5: Delivering telerehabilitation

We would like to understand more about how you currently feel about telerehabilitation (including delivery of rehabilitation and psychological support). There are no right or wrong answers. Please select the response that relates most closely to your current situation and experience.

Please don't select more than 1 answer(s) per row.

	Strongly Agree	Agree	Somewhat Agree	Neither Agree not Disagree	Somewhat Disagree	Disagree	Strongly Disagree
I am aware of the content of an effective telerehabilitation programme	☐	☐	☐	☐	☐	☐	☐
I am aware of the objectives of a telerehabilitation programme	☐	☐	☐	☐	☐	☐	☐
I know what my responsibilities are, with regard to delivering a therapy session using telerehabilitation	☐	☐	☐	☐	☐	☐	☐
I know how to use telerehabilitation	☐	☐	☐	☐	☐	☐	☐
I know when to use telerehabilitation	☐	☐	☐	☐	☐	☐	☐
I have received training regarding how to deliver telerehabilitation	☐	☐	☐	☐	☐	☐	☐

I have the skills needed to deliver a telerehabilitation programme	☐	☐	☐	☐	☐	☐	☐
I have been able to practice using telerehabilitation	☐	☐	☐	☐	☐	☐	☐
Delivering therapy sessions using telerehabilitation is part of my role	☐	☐	☐	☐	☐	☐	☐
It is my responsibility to delivery therapy sessions using telerehabilitation protocols	☐	☐	☐	☐	☐	☐	☐
Delivering therapy sessions using telerehabilitation is consistent with other aspects of my job	☐	☐	☐	☐	☐	☐	☐

We would like to understand more about your confidence of delivering telerehabilitation and whether you feel able to deliver rehabilitation and/or psychological support in this way. Please select the response that relates most closely to your current situation and experience.

Please don't select more than 1 answer(s) per row.

	Strongly Agree	Agree	Somewhat Agree	Neither Agree not Disagree	Somewhat Disagree	Disagree	Strongly Disagree

I am confident that I can plan and deliver therapy sessions with my patients using telerehabilitation protocols	☐	☐	☐	☐	☐	☐	☐
I am capable of planning and delivering telerehabilitation, even when little time is available	☐	☐	☐	☐	☐	☐	☐
I have the confidence to plan and delivery therapy using telerehabilitation even when other professionals I work with are not doing this	☐	☐	☐	☐	☐	☐	☐
I have the confidence to plan and deliver therapy using telerehabilitation even when the patients who attend the service are not receptive	☐	☐	☐	☐	☐	☐	☐
I have personal control over planning and delivering therapy using telerehabilitation	☐	☐	☐	☐	☐	☐	☐

For me, planning and delivering therapy using telerehabilitation is easy	☐	☐	☐	☐	☐	☐	☐
In uncertain times, when I plan and deliver therapy using telerehabilitation I usually expect that things will work out okay	☐	☐	☐	☐	☐	☐	☐
When I plan and deliver therapy using telerehabilitation, I feel optimistic about my job in the future	☐	☐	☐	☐	☐	☐	☐
I do not expect anything will prevent me from using telerehabilitation to deliver therapy to my patients	☐	☐	☐	☐	☐	☐	☐

We would like to understand more about your beliefs and motivations for using telerehabilitation (including delivery of rehabilitation and psychological support). *There are no right or wrong answers. Please select the response that relates most closely to your current situation and experience.*

Please don't select more than 1 answer(s) per row.

	Strongly Agree	Agree	Somewhat Agree	Neither Agree not Disagree	Somewhat Disagree	Disagree	Strongly Disagree

I believe delivering each of my patients' sessions using telerehabilitation will lead to benefits for the patients who attend the service	☐	☐	☐	☐	☐	☐	☐
I believe delivering each of my patients' sessions using telerehabilitation will benefit public health (ie. health of the whole population)	☐	☐	☐	☐	☐	☐	☐
In my view, using telerehabilitation to deliver each of my patients' sessions is useful	☐	☐	☐	☐	☐	☐	☐
In my view, using telerehabilitation to deliver each of my patients' sessions is worthwhile	☐	☐	☐	☐	☐	☐	☐

I get recognition from management at the organisation where I work, when I use telerehabilitation to deliver my patients' sessions	☐	☐	☐	☐	☐	☐	☐
When I use telerehabilitation to deliver my patients' sessions, I get recognition from my colleagues	☐	☐	☐	☐	☐	☐	☐
When I use telerehabilitation to deliver my patients' sessions, I get recognition from those whom it impacts	☐	☐	☐	☐	☐	☐	☐
I intend to apply telerehabilitation protocols to each/every one of my patients' sessions	☐	☐	☐	☐	☐	☐	☐
I will definitely apply telerehabilitation protocols to each/every one of my patients' sessions	☐	☐	☐	☐	☐	☐	☐

I have a strong intention to apply telerehabilitation protocols to each/every one of my patients' sessions	☐	☐	☐	☐	☐	☐	☐

We would like to understand more about how you currently feel about telerehabilitation and the support you receive to deliver rehabilitation in this way. *There are no right or wrong answers. Please select the response that relates most closely to your current situation and experience.*

Please don't select more than 1 answer(s) per row.

	Strongly Agree	Agree	Somewhat Agree	Neither Agree nor Disagree	Somewhat Disagree	Disagree	Strongly Disagree
Compared to my other tasks, planning how and delivering my therapy using telerehabilitation is a higher priority on my agenda	☐	☐	☐	☐	☐	☐	☐
Compared to my other tasks, planning how and delivering my therapy using telerehabilitation is an urgent item on my agenda	☐	☐	☐	☐	☐	☐	☐

I have clear goals related to using telerehabilitation to deliver each of my patients' sessions	☐	☐	☐	☐	☐	☐	☐
Using telerehabilitation to deliver each of my patients' sessions is something I do automatically	☐	☐	☐	☐	☐	☐	☐
In the organisation I work, all necessary resources are available to allow me to deliver my planned therapy using telerehabilitation	☐	☐	☐	☐	☐	☐	☐
I have support from the management of the organisation to deliver my planned therapy using telerehabilitation	☐	☐	☐	☐	☐	☐	☐

The management of the organisation I work for are willing to listen to any problems I have when delivering my planned therapy using telerehabilitation	☐	☐	☐	☐	☐	☐	☐
The organisation I work for provides the opportunity for training to deliver my planned therapy using telerehabilitation	☐	☐	☐	☐	☐	☐	☐
The organisation I work for provides sufficient time for me to deliver my planned therapy using telerehabilitation	☐	☐	☐	☐	☐	☐	☐
People who are important to me think that I should deliver therapy using telerehabilitation	☐	☐	☐	☐	☐	☐	☐
People whose opinion I value would approve of me delivering therapy using telerehabilitation	☐	☐	☐	☐	☐	☐	☐

I can count on support from colleagues whom I work with when things get tough with delivering therapy sessions using telerehabilitation	☐	☐	☐	☐	☐	☐	☐
Colleagues whom I work with are willing to listen to the problems I have when delivering therapy sessions using telerehabilitation	☐	☐	☐	☐	☐	☐	☐

We would like to understand more about how you currently feel about telerehabilitation. *There are no right or wrong answers. Please select the response that relates most closely to your current situation and experience.*

Please don't select more than 1 answer(s) per row.

	Strongly Agree	Agree	Somewhat Agree	Neither Agree nor Disagree	Somewhat Disagree	Disagree	Strongly Disagree
I am able to deliver therapy using telerehabilitation without feeling anxious	☐	☐	☐	☐	☐	☐	☐
I am able to deliver therapy using telerehabilitation without feeling distressed or upset	☐	☐	☐	☐	☐	☐	☐

I am able to deliver therapy using telerehabilitation, even when I feel stressed	☐	☐	☐	☐	☐	☐	☐
I have a detailed plan of how I will deliver therapy using telerehabilitation	☐	☐	☐	☐	☐	☐	☐
I have a detailed plan of how I will deliver therapy using telerehabilitation when patients who usually attend the service are not receptive	☐	☐	☐	☐	☐	☐	☐
I have a detailed plan of how I will deliver therapy using telerehabilitation when there is little time	☐	☐	☐	☐	☐	☐	☐
It is possible to adapt how I will deliver therapy using telerehabilitation to meet my needs as a rehabilitation therapist/psychologist	☐	☐	☐	☐	☐	☐	☐
Delivering therapy using telerehabilitation is compatible with other aspects of my job	☐	☐	☐	☐	☐	☐	☐

Page 6: Final page

Thank you for taking part in this study.

We appreciate you taking the time to complete the questionnaire. Your feedback and experiences will help us to further adapt the ROWTATE intervention ahead of the trial.

http://www.ROWTATE.org.uk

Supplementary File 3 - Participant semi-structured pre-intervention interview topic guide (Therapists)

Version 1.0 | Date 08.06.2020
IRAS no. 265431

Multicentre Research Programme to Enhance Return to Work after Trauma (ROWTATE) – Feasibility of helping patients return to work during the Covid-19 pandemic.

(Chief Investigators: Professor Denise Kendrick and Dr Kate Radford)

General:

1. Have you had to adapt clinical practice during COVID-19 to deliver services remotely? If so, how?
2. What devices and online platforms have you been using?
3. Have you experienced any issues in delivering tele-rehabilitation or telepsychology?
4. What are the main factors that could help or hinder the delivery of ROWTATE via tele-rehabilitation?
5. What additional support might you require?

Theoretical Domains Framework specific questions:

1. ***Knowledge***
   - What experience do you have of delivering tele-rehabilitation or telepsychology?
   - Have you had any formal or informal training in doing this?
     - Via professional organisation, NHS Trust, online (e.g. YouTube)
   - Are there any gaps in your knowledge?

2. ***Social/professional role and identity***
   - To what extent is tele-rehabilitation or telepsychology part of your professional role?

3. ***Skills***
   - How easy or difficult would you find delivering the ROWTATE intervention via tele-rehabilitation?
   - Do you think there are any particular skills required to do this?
   - What additional training would you need?
   - Are there any aspects of vocational rehabilitation that you might find difficult to deliver remotely?
     - Workability assessments?
     - Cognitive assessments?
     - Work hardening?
     - Work site assessments?

4. ***Beliefs about capabilities***
   - How confident are you that you could deliver the ROWTATE intervention via tele-rehabilitation?
   - If not very confident, is there any specific training that could help increase your confidence?

5. ***Beliefs about consequences***
   - Do you think there would be any benefits or disadvantages of delivering the ROWTATE intervention via tele-rehabilitation or telepsychology?

6. Environmental context and resources

- What work factors or resources may help or hinder the delivery of ROWTATE via tele-rehabilitation?
    - Access to work phone, cost of calls, free Wi-Fi, security issues?
    - Support from NHS Trust/manager/IT support?
    - Hardware/software/licences?
- Are there systems in place at work to support remote delivery of the intervention?
    - NHS Trust policies/guidelines
    - Training
    - Access to the internet

Questions specific to training:

1. Have you participated in any form of online training?
    - If yes, what platforms did this use? (Zoom, MS Teams, Skype, Adobe connect, etc.)

2. What training do you think you would need to provide the ROWTATE intervention remotely?
    - Training in using devices and/or software for remote delivery?
    - Training in online security/data protection/online safety?
    - Training in providing specific aspects of vocational rehabilitation remotely e.g. workability assessments, cognitive assessments, work hardening, work site assessments
    - Any other training needs you can think of?

Thinking now about the ROWTATE training for face-to-face delivery you received earlier in the year:

3. How do you think we can adapt the ROWTATE training you received to be able to provide it remotely in the future?

4. Assuming the ROWTATE training can be adapted and delivered online using a combination of lectures and interactive workshops, what would be your preference for delivery format?
    - 4 half day training sessions with breaks?
    - A series of brief (1-2 hour) daily/ bi-weekly training sessions over a 1-2 week period
    - What times of day would work best for you? (e.g. early morning 8.30-10am, mid morning 10.30am-12pm, lunchtimes 12-1.30pm, afternoon 1.30-3pm, evenings 4-6pm)

5. From your memory of the ROWTATE training, which aspects of the training do you think would work well remotely?
    - Group work, presentations, discussions etc.
    - Working with PPI, meeting people from other centres, meeting study team, sharing knowledge.

6. Which aspects of the training might not work so well remotely? Why?

7. In the ROWATE training we had an actor play the part of a patient and you completed individual and team tasks. Assuming we can create an online version of the team based assessment (TOSCE), how do you think we could do the individual and team tasks remotely?

8. What online platforms would you prefer the training to be provided using and why?

9. Can you foresee any technological difficulties for you if the training was provided remotely?

10. Is there anything else you would like to tell us about adapting the ROWTATE training?

Supplementary File 4 - Participant semi-structured post-intervention interview topic guide: Trauma survivor

Version 4.0 Date 11.02.2021
IRAS no. 265431

Multicentre Research Programme to Enhance Return to Work after Trauma (ROWTATE) – Feasibility of helping patients return to work during the Covid-19 pandemic

Chief Investigators: Dr Kate Radford & Professor Denise Kendrick

Over the past few months, you have been receiving help/advice/support from an occupational therapist (and clinical psychologist if relevant) to help you return to work as part of the ROWTATE study. These questions are about the help/advice/support you have received.

General:

1. Can you start by telling us about your experience of the help/advice/support you have received as part of the ROWTATE study?
2. Can you tell us about your experience of your employer taking part in ROWTATE?
3. Can you tell us about your experience of working with your occupational therapist (OT)/clinical psychologist (CP)?

Theoretical Framework of Acceptability:

1. *Affective attitude*
   - How did you/do you feel about the help/advice/support you received from the OT (and CP if relevant)?

2. *Burden*
   - How much of an effort is/was it for you to engage with the help/advice/support you received?

3. *Ethicality*
   - Was there any help/advice/support that did not feel right to you, or that you disagreed with?
   - Did the help/advice/support have any negative consequences for you?

4. *Intervention coherence*
   - What help/advice/support have you received for the OT (and CP if relevant)?
   - What do you think the help/advice/support is trying to achieve?
   - How easy has it been to act on the advice/support you have been given?

5. *Opportunity costs*
   - What has it cost you in terms of money, time or other resources to engage with the help/advice/support from the OT (and CP if relevant)?

6. *Perceived effectiveness*
   - How well do you think the help/advice/support is working/has worked for you?

7. *Self-efficacy*
   - How confident are you that you can act on the help/advice/support from the OT (and CP if relevant)?
   - What was that advice?
   - Why was the support/help/advice difficult/easy to follow?

Question relating to remote delivery of the intervention:

1. What do you think about getting the help/advice/support from the OT (and CP if relevant) via phone/videocalls/other remote methods?
2. What were the challenges to getting the help/advice/support in this way?
3. How do you think getting help/advice/support by phone/videocall etc. affected your relationship with your OT/CP?
4. Overall, how well did the technology work for you?
5. If applicable, what difficulties did you encounter and how could they be overcome in the future?
6. Was there any technology/devices/software that would have helped but did not have?
7. If you had a choice in future to have help/advice/support provided face-face or by phone/videocalls/other remote methods, which would you prefer and why?
8. Is there anything else you would like to tell us about the help/advice/support you received by phone/videocall/other remote methods?

COVID-19 specific questions:

1. Has the COVID-19 pandemic affected your return to work in any way? If so, how?
   a. *Contact/support from others (health professionals, employer)*
   b. *Access to resources/equipment (e.g., computer)*
   c. *Transport to/from workplace*
   d. *Returning to work sooner than ideal (e.g., essential worker, needed money)*
   e. *Started new temporary role (e.g., supermarket, NHS volunteer, childcare)*
   f. *Reduced hours, or not able to work (graded return postponed, furloughed, shielding/self-isolating, made redundant)*

2. Have you experienced any unexpected benefits as a result of the COVID-19 pandemic?
   - *More regular contact with health professionals/employers*
   - *Provision of support (e.g., information, advice, equipment, financial)*
   - *Reduction in fatigue (e.g., reduced travel time etc)*

<u>Prompts:</u>

- Tell me more about that …
- How did that feel?
- Can you give me an example of …
- What do you mean by …

Supplementary File 5 - Participant semi-structured post-intervention interview topic guide: Therapists

Version 4.0 Date 11.02.2021
IRAS no. 265431

Multicentre Research Programme to Enhance Return to Work after Trauma (ROWTATE) – Feasibility of helping patients return to work during the Covid-19 pandemic

Chief Investigators: Dr Kate Radford & Professor Denise Kendrick

Over the past few months, you have been delivering a return to work intervention as part of the ROWTATE study. These questions are about the help/advice/support you have provided.

General:

- Tell us about your experience of providing help/advice/support to patients and their employers as part of the ROWTATE study.
- What did you find worked best?
- What did you find did not work well?
- If you could make changes to the help/advice/support in ROWTATE, what would you change and why?

Theoretical Framework of Acceptability:

1. *Affective attitude*
   - How do you feel about the help/advice/support you have provided during the ROWTATE study?
2. *Burden*
   - How much of an effort was it for you to provide the help/advice/support during the ROWTATE study?
3. *Ethicality*
   - To what extent does/did the help/advice/support you provided during the ROWTATE study feel right to you, or fit with the values of your employing organisation?
   - Did providing the help/advice/support during the ROWTATE study have any negative consequences for you, your patients or their employers?
4. *Intervention coherence*
   - What support/help/advice have you provided for patients and employers during the ROWTATE study?
   - What is/was the help/advice/support you provide(d) in the ROWTATE study trying to achieve?
   - How well do you think patients have been able to act on your advice?
5. *Opportunity costs*
   - What has it cost you in terms of money, time, other resources to provide help/advice/support during the ROWTATE study?
6. *Perceived effectiveness*
   - How well do you think the help/advice/support you have provided during the ROWTATE study is working/has worked?
7. *Self-efficacy*
   - How confident are you that you can provide the help/advice/support (as part of the ROWTATE study) to patients and their employers?

Question relating to remote delivery of the intervention:

1. What do you think about providing the help/advice/support during the ROWTATE study via phone/videocalls/other remote methods?
   a. What challenges did find in doing this?

2. Are there any groups of patients that you think remote help/advice/support is less suitable for?
   a. Why is this?
   b. How might difficulties with remote provision with these patients be overcome in the future?

3. How do you think providing help/advice/support remotely affected your relationship with your patient?
   a. (If any negative impacts mentioned) how do you think this affected how well your patient engaged with the help/advice/support?

4. Did you provide any help/advice/support face-face with patients or employers?
   a. Why did you choose face-face for this help/advice/support?

5. Overall, how well did the technology work for providing help/advice/support remotely?
   a. (If experienced any difficulty with technology) What difficulties did you encounter and how could they be overcome in the future?

6. How well did the ROWTATE training prepare you to provide help/advice/support for patients and employers remotely?

7. If you had a choice in future to provide the ROWTATE study help/advice/support face-face or by phone/videocalls/other remote methods, which would you prefer and why?

8. Is there anything else you would like to tell us about your experiences of providing the ROWTATE help/advice/support remotely?

COVID-19 specific questions

1. **Do you feel that the COVID-19 pandemic has impacted on your patient's ability to return to work?**
   a. How has it impacted on their return to work?
   b. What experience do you have of patients losing their jobs because of COVID? If applicable, how has this affected the intervention you provided?
   c. Has the patient's employer been supportive? If not, have you managed to overcome challenges and how?

2. **Do you feel that the COVID-19 pandemic has impacted on patients in other ways?**
   a. Are patients requiring more psychological support during their recovery and return to work?
   b. Are patients experiencing more financial challenges?
   c. Are there any other effects of COVID-19 that have impacted on someone's ability return to work?

3. **Has the COVID-19 pandemic affected your ability to deliver the intervention in any way?**
   a. Ability to contact necessary stakeholders without needing to book face-to-face appointment/more flexible (e.g. patients, employers).
   b. Access to resources/equipment (e.g., computer to deliver sessions).
   c. Challenges with assessing patients remotely, risks associated with face-to-face sessions.
   d. Employers having to furlough staff, not being supportive, patients having to self-isolate etc.

4. **Have you experienced any unexpected benefits in delivering the intervention during the COVID-19 pandemic?**
   a. Ability to have more regular and flexible contact with patients/employers.
   b. Reduction in time spent doing additional non-therapy activities (e.g. reduced travel time etc.)
   c. Patients not feeling anxious about attending face-to-face appointments, patients not having to travel to appointments so reduced fatigue etc.
